# Cerebral Immunohistochemical Characterization of the H_2_S and the Oxytocin Systems in a Porcine Model of Acute Subdural Hematoma

**DOI:** 10.3389/fneur.2020.00649

**Published:** 2020-07-07

**Authors:** Nicole Denoix, Tamara Merz, Sarah Unmuth, Andrea Hoffmann, Ester Nespoli, Angelika Scheuerle, Markus Huber-Lang, Harald Gündel, Christiane Waller, Peter Radermacher, Oscar McCook

**Affiliations:** ^1^Clinic for Psychosomatic Medicine and Psychotherapy, Ulm University Medical Center, Ulm, Germany; ^2^Institute for Anesthesiological Pathophysiology and Process Engineering, Ulm University Medical Center, Ulm, Germany; ^3^Department of Neurology, Molecular and Translational Neuroscience, Ulm University, Ulm, Germany; ^4^Department of Neuropathology, Institute for Pathology, Ulm University Medical Center, Ulm, Germany; ^5^Institute for Clinical and Experimental Trauma Immunology, Ulm University Medical Center, Ulm, Germany; ^6^Department of Psychosomatic Medicine and Psychotherapy, Nuremberg General Hospital, Paracelsus Medical University, Nuremberg, Germany

**Keywords:** traumatic brain injury, albumin, cystathionine-ɤ-lyase, barrier dysfunction, edema, complement system, oxytocin receptor, resuscitation

## Abstract

The hydrogen sulfide (H_2_S) and the oxytocin/oxytocin receptor (OT/OTR) systems interact in trauma and are implicated in vascular protection and regulation of fluid homeostasis. Acute brain injury is associated with pressure-induced edema formation, blood brain barrier disruption, and neuro-inflammation. The similarities in brain anatomy: size, gyrencephalic organization, skull structure, may render the pig a highly relevant model for translational medicine. Cerebral biomarkers for pigs for pathophysiological changes and neuro-inflammation are limited. The current study aims to characterize the localization of OT/OTR and the endogenous H_2_S producing enzymes together with relevant neuro-inflammatory markers on available porcine brain tissue from an acute subdural hematoma (ASDH) model. In a recent pilot study, anesthetized pigs underwent ASDH by injection of 20 mL of autologous blood above the left parietal cortex and were resuscitated with neuro-intensive care measures. After 54 h of intensive care, the animals were sacrificed, the brain was removed and analyzed via immunohistochemistry. The endogenous H_2_S producing enzymes cystathionine-ɤ-lyase (CSE) and cystathionine-β-synthase (CBS), the OTR, and OT were localized in neurons, vasculature and parenchyma at the base of sulci, where pressure-induced injury leads to maximal stress in the gyrencephalic brain. The pathophysiological changes in response to brain injury in humans and pigs, we show here, are comparable. We additionally identified modulators of brain injury to further characterize the pathophysiology of ASDH and which may indicate future therapeutic approaches.

## Introduction

Recently we described a long-term (54 h) resuscitated porcine model of acute subdural hematoma (ASDH)-induced acute brain injury, which comprised elevated intracranial pressure (ICP), and morphological damage without major neurological dysfunction, due to the neuro-intensive care maintenance of cerebral perfusion pressure (CPP) and tissue oxygen (PbtO_2_) (1).

In spite of many promising results with rodent acute brain injury models, translation into benefits for the clinic seems problematic (2). This may at least in part be due to the fact that rodents have lissencephalic brains, whereas humans have gyrencephalic brains (3, 4). Gyrencephalic brains are more susceptible to pressure-induced acute brain injury, because in the gyrencephalic brain the maximal mechanical pressure occurs at the base of the sulci, whereas the lissencephalic brain structure of the rodent brain allows for the pressure elevation to be distributed more evenly (2, 5). The pig as a model organism entails various advantageous similarities to humans regarding the brain: size, gyrencephalic neuroanatomical organization, skull structure, and proportion of white matter to gray matter (2, 5). The skull and tentorium cerebelli of pigs and humans have similar rigid characteristics in contrast to rodents, which only have a “vestigial connective tissue membrane” (2). The rigidity of the tentorium cerebelli, which separates the cerebrum from the cerebellum, is significant in confining the injury-induced edema formation and increase in ICP following acute brain injury, which mostly occurs in the cerebrum in humans and pigs (2). Furthermore, the adult pig and human brain are composed of 60% white matter, in contrast to <12% in the adult rat brain (5). This may be crucial for translation since white matter is more prone to develop edema and thus aggravates the pressure-induced trauma in the cerebrum (2, 6).

Hydrogen sulfide (H_2_S), a gaseous mediator, and the oxytocin (OT)/oxytocin receptor (OTR) system are implicated in fluid homeostasis and may play a role in edema (7). Their interaction has been reported in trauma (8, 9). In our porcine ASDH pilot study, markers of blood brain barrier (BBB) dysfunction and oxidative and nitrosative stress, were significantly elevated in the ipsilateral side, i.e., the side of ASDH induction. Since both H_2_S and the OT/OTR systems have been reported to play a role as anti-oxidants and in vascular protection, they could be relevant players in ASDH (10–13).

Immunohistochemical studies in porcine acute brain injury are limited (2, 14–20). In particular, the cell-specific cerebral localization and distribution of the endogenous H_2_S producing enzymes, OT and OTR in association with mediators of injury are not reported. Thus, information on protein localization in the global landscape of the brain, which is not assessable otherwise, is clearly lacking (21).

Therefore, the aim of the present study is to further characterize, immunohistochemically, the spatial protein expression of OTR, OT and the H_2_S producing enzymes and assessing potential mediators for porcine acute brain injury in the available material from our ASDH pilot study (1).

## Materials and Methods

The study was approved by the Federal Authorities for Animal Research and the local Animal Care Committee (Reg.-Nr. 1316, date of approval June 20, 2016). All experiments were conducted in adherence with the National Institute of Health Guidelines on the Use of Laboratory Animals and the European Union “Directive 2010/63/EU on the protection of animals used for scientific purposes.” This is an immunohistochemical study performed on available brain sections from a recently published porcine model of ASDH (1): 5 female and 4 male (castrated) anesthetized Bretoncelles-Meishan-Willebrand pigs (age 11 months, 65 kg). IHC allowed us to study and visualize the nuclear or cytoplasmic protein localization in the global landscape of the brain. In contrast to techniques which only use homogenized tissue, e.g., western blot, immunohistochemistry (IHC) allowed for detecting the spatial expression patterns and distinguishing between tissue and blood, a common confounding factor in homogenized tissue (22). We used a colorimetric detection system which allows us to visualize the tissue architecture and cellular morphology (limited in immunofluorescence), e.g., vacuolization or necrosis.

Anesthesia, surgical procedures and resuscitation were described in detail previously (1) ASDH was induced by injection of 20 mL of autologous blood via a subdural catheter above the left parietal cortex. Therefore, the skull was exposed and a craniotomy was performed by drilling a hole over the left and right parietal cortex and a small incision of the dura was made. According to the 3R principle the right hemisphere was also instrumented to avoid the need for additional sham experiments. Microdialysis catheters and multimodal brain monitoring probes were inserted in both hemispheres of brain parenchyma (1).

Two hours after ASDH induction, resuscitation was initiated: CPP was titrated to baseline pressure (if not sufficient norepinephrine was used), neuro-intensive care measures were in accordance with the current guidelines of traumatic brain injury (TBI) management in an effort to further improve clinical translation of novel therapeutic interventions (23). After 54 h of intensive care, anesthesia was further deepened and pigs were sacrificed via injection of potassium chloride.

### Immunohistochemistry

Immediately after termination of the experiment the brain was removed, cut sagittally to separate the hemispheres and fixed in 4% formalin. After 6 days of fixation (fixation identical for all samples) the brain was cut from frontal to occipital into consecutive 4 mm thick macroscopic sections (13–17 sections in total), dehydrated, and embedded in paraffin blocks (1).

Available brain sections for further IHC included frontal, medial and occipital (limited, brainstem and cerebellum) regions from the ipsi- and contralateral side. Paraffin sections were cut (3–5 μm), deparaffinized in xylene, and rehydrated with a graded series of ethanol and deionized water. Heat-induced antigen retrieval was performed by bringing the samples to a boil in a microwave oven in a 10 mM citrate solution (pH 6) and cooling back to room temperature. All the following steps were performed at room temperature. Blocking with normal goat serum (10%) for 20–30 min was performed before 1 h primary antibody incubation with: endogenous H_2_S producing enzymes anti-cystathionine-ɤ-lyase (CSE) (Protein Tech, 12217-1-AP) and anti-cystathionine-β-synthase (CBS) (Protein Tech, 14787-1-AP), anti-OT (Millipore, AB911), anti-OTR [Protein Tech, 23045-1-AP (10)]. Further mediators of injury were investigated by using the following primary antibodies: anti-pig albumin (Alb) (Abcam, ab79960) (1); anti-aquaporin 4 (AQP4) (Protein Tech, 16473-1-AP); anti-neuronal nuclei (NeuN) (Cell signaling, 24307) as a marker for neurons; anti-complement component 5a receptor (C5aR) (Protein Tech, 21316-1-AP); anti-induced complement component 3 (iC3) (Hycult biotech, HM2168); anti-complement component 1 Q subcomponent-binding protein (C1QBPBP) (Protein Tech, 24474-1-AP); anti-interleukin 6 receptor (Il-6R) (Protein Tech, 23457-1-AP); anti-tumor necrosis factor receptor 1 and 2 (TNFR1 (Protein Tech, 21574-1-AP) and TNFR2 (Protein Tech, 19272-1-AP). Sections of ipsi- and contralateral brain specimens were analyzed concurrently, and negative controls were performed (examples see Figure S1). Since the IHC detection of OTR in the brain has proven to be a particular challenge in the past, the specificity of the OTR antibody used in this study was validated by both negative controls [knock out (9)] and positive controls, western blot and immunoprecipitation (Proteintech, methods description and results in Figure S2). The specificity of the antibodies has been additionally confirmed in NCBI BLAST searches (courtesy of the U.S. National Library of Medicine). The BLAST “finds regions of similarities between biological sequences, and compares protein sequences to sequence databases.” We compared immunogen sequences of the used antibodies to the sus scrofa database (see Table 1). When the immunogen sequence was not available we used the full length protein sequence of the species the antibody was made to, in this case human. A query cover above 90% and homology above 60% were investigated, when there was no significant homology to any other protein.

**Table 1:** Protein BLAST search for primary antibodies (anti-human) to sus scrofa.

**Primary antibody (Source, catalog no., RRID)**	**Host species**	**Query cover (%)**	**Homology (%)**	**Immunogen sequence**	**Concentration used for IHC**
anti-CSE (Protein Tech, 12217-1-AP, RRID:AB_2087497)	Rabbit Polyclonal	100	87.05	Gamma cystathionase fusion protein Ag2872	1:200
anti-CBS (Protein Tech, 14787-1-AP, RRID:AB_2070970)	Rabbit Polyclonal	100	90.46	CBS fusion protein Ag6437	1:100
anti-OT (Millipore, AB911, RRID:AB_2157629)	Rabbit Polyclonal	100	100	CYIQNCPLG (Synthetic oxytocin (Sigma) conjugated to thyroglobulin)	1:500
anti-OTR (Protein Tech, 23045-1-AP, RRID:AB_2827425)	Rabbit Polyclonal	92	83.05	Oxytocin Receptor fusion protein Ag19074	1:100
anti-AQP4 (Protein Tech, 16473-1-AP, RRID:AB_2827426)	Rabbit Polyclonal	100	94.83	Aquaporin 4 fusion protein Ag9561	1:2,000
anti-NeuN (Cell signaling, 24307, RRID:AB_2651140)	Rabbit Monoclonal	98	97.08	human full length protein: Swiss-Prot Acc.: A6NFN3	1:400
anti-C5aR (Protein Tech, 21316-1-AP, RRID:AB_10733105)	Rabbit Polyclonal	100	65.00	C5aR fusion protein Ag15968	1:100
anti-iC3 (Hycult biotech, HM2168, RRID:AB_10130959)	Mouse Monoclonal	100	75.06	human full length protein: UniProtKB - P01024 (CO3_HUMAN)	1:50
anti-C1QBPBP (Protein Tech, 24474-1-AP, RRID:AB_2827427)	Rabbit Polyclonal	100	85.11	C1QBP fusion protein Ag19773	1:100
anti-Il-6R (Protein Tech, 23457-1-AP, RRID:AB_2827428)	Rabbit Polyclonal	100	77.21	IL-6R fusion protein Ag18263	1:100
anti-TNFR1 (Protein Tech, 21574-1-AP, RRID:AB_10734433)	Rabbit Polyclonal	100	69.30	TNFR1 fusion protein Ag16112	1:100
anti-TNFR2 (Protein Tech, 19272-1-AP, RRID:AB_10640674)	Rabbit Polyclonal	100	67.73	TNFR2 fusion protein Ag5866	1:100

Detection of primary antibodies was performed according to the manufacturer's instructions using the Dako REAL detection system (based on alkaline phosphatase conjugated secondary antibodies: anti-mouse, anti-rabbit), and visualized with red chromogen followed by counterstaining with Mayer's hematoxylin (1, 12, 24). Slides were visualized using a Zeiss Axio Imager A1 microscope with 2.5X, 10X, 20X. and 40X objectives.

## Results

The pictures in the following figures are representative examples of all the IHC experiments for each protein of interest, performed on tissue from various animals. The exact number of animals are given in the figure legends.

**Figure 1:**
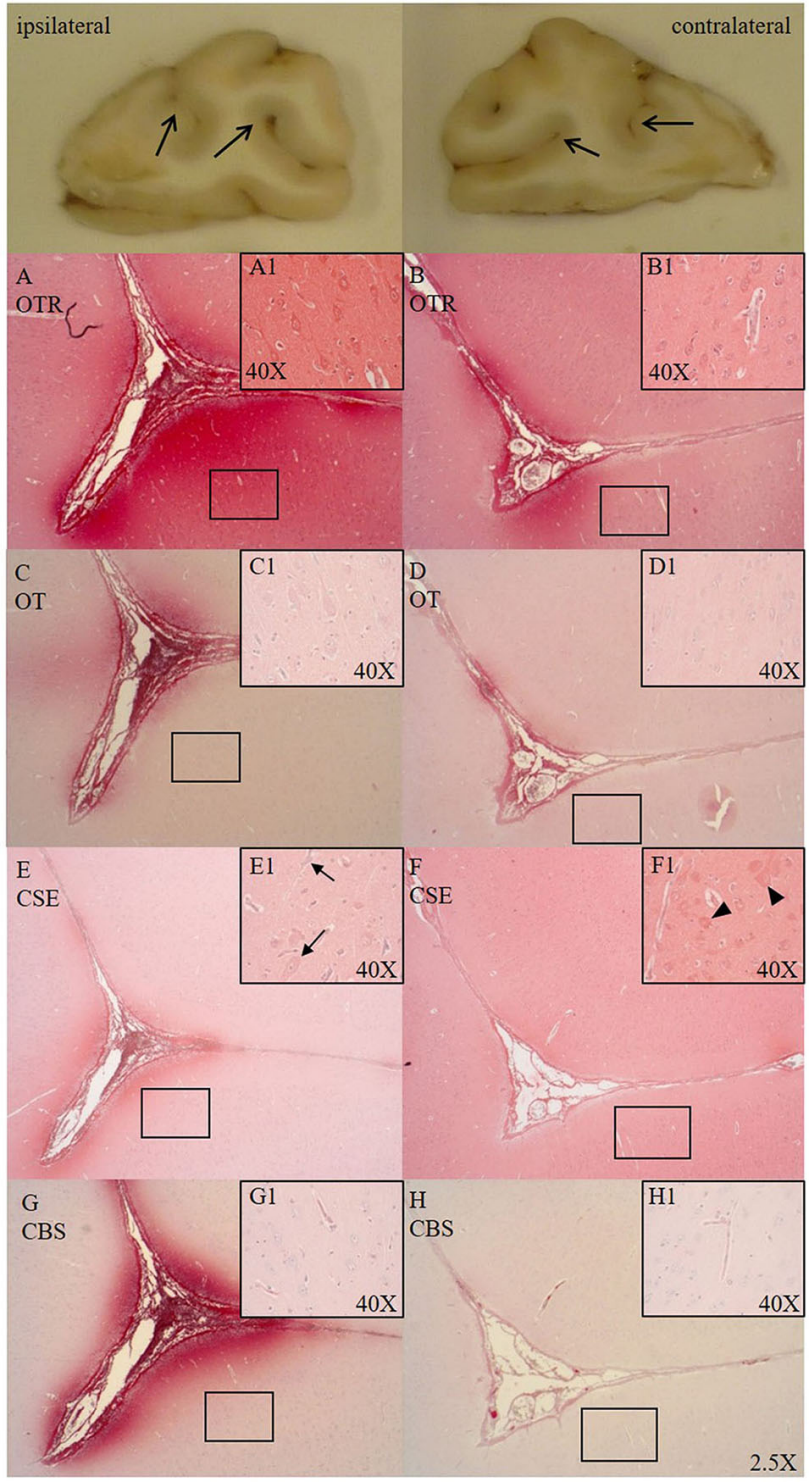
Comparison of OTR (**A,B**, *n* = 6), OT (**C,D**, *n* = 7), CSE (**E,F**, *n* = 7), and CBS (**G,H**, *n* = 6) expression in matching consecutive sections of a sulcus of the frontal cortex [ipsilateral **(A,C,E,G)** vs. contralateral side **(B,D,F,H)**]. Macroscopic exemplary sections are shown at the top (open arrows pointing to sulci). Ipsilateral OTR expression in the pial vasculature and glia limitans with blushing effect in the parenchyma surrounding the base of the sulcus **(A)**, cortical neurons magnified in **(A1)**, less pronounced OTR expression on the contralateral site **(B)**, cortical neurons magnified in **(B1)**. OT expression is more limited than OTR expression but shows the same expression pattern as OTR in the ipsi- (**C**, magnified in **C1**) and contralateral side (**D**, magnified in **D1**). In the ipsilateral side CSE is pronouncedly expressed surrounding the sulcus and negative in the parenchyma moving away from the sulcus **(E)** and shows little to no expression in cortical neurons, with nuclear counter-staining with hematoxylin (black arrows magnified in **E1**). CSE is expressed in the pial vasculature and is evenly distributed in parenchyma of the contralateral side around the sulcus **(F)** with (red) CSE-positive cortical neurons (black arrow heads magnified in **F1**). In the ipsilateral side CBS shows pronounced expression surrounding the sulcus **(G)** and is not expressed in cortical neurons (magnified in **G1**). CBS expression in the contralateral side is weak **(H)** and present in the microvasculature (magnified in **H1**). OTR: oxytocin receptor; OT, oxytocin; CSE, cystathionine-ɤ-lyase; CBS, cystathionine-β-synthase.

Cytoplasmic OTR expression was found in cortical neurons in both hemispheres of the frontal cortex (see Figures 1A1,B1). OTR and OT expression was present in the parenchyma around the sulci of the ipsilateral side (see Figures 1A,C). Specifically, we observed an OTR positive lining around the nucleus of small cells in the white matter. Sections from the ASDH induction site showed OTR expression in and around the parenchyma of necrotic areas (associated with loss of cellular architecture/vacuolization). Hippocampal neurons and granular neurons showed OTR and OT expression, primarily in the cytoplasm. Perivascular swelling was associated with more parenchymal OTR expression (see Figure 1A1).

CSE was expressed in cortical neurons (cytoplasmic) of the frontal cortex in the contralateral side (see Figure 1F1) and low in the ipsilateral side (see Figure 1E) confined to the sulci. CSE was also expressed in hippocampal neurons and granular neurons. At the injury site, CSE was present in necrotic areas where parenchymal bleeding was present and tissue architecture was disrupted (CSE potentially coming from the circulation/blood). There was little CBS expression in the parenchyma of the contralateral side (see Figure 1H). In the ipsilateral side CBS was strongly expressed around the sulcus (see Figure 1G) and not expressed in cortical neurons (see Figure 1G1).

**Figure 2:**
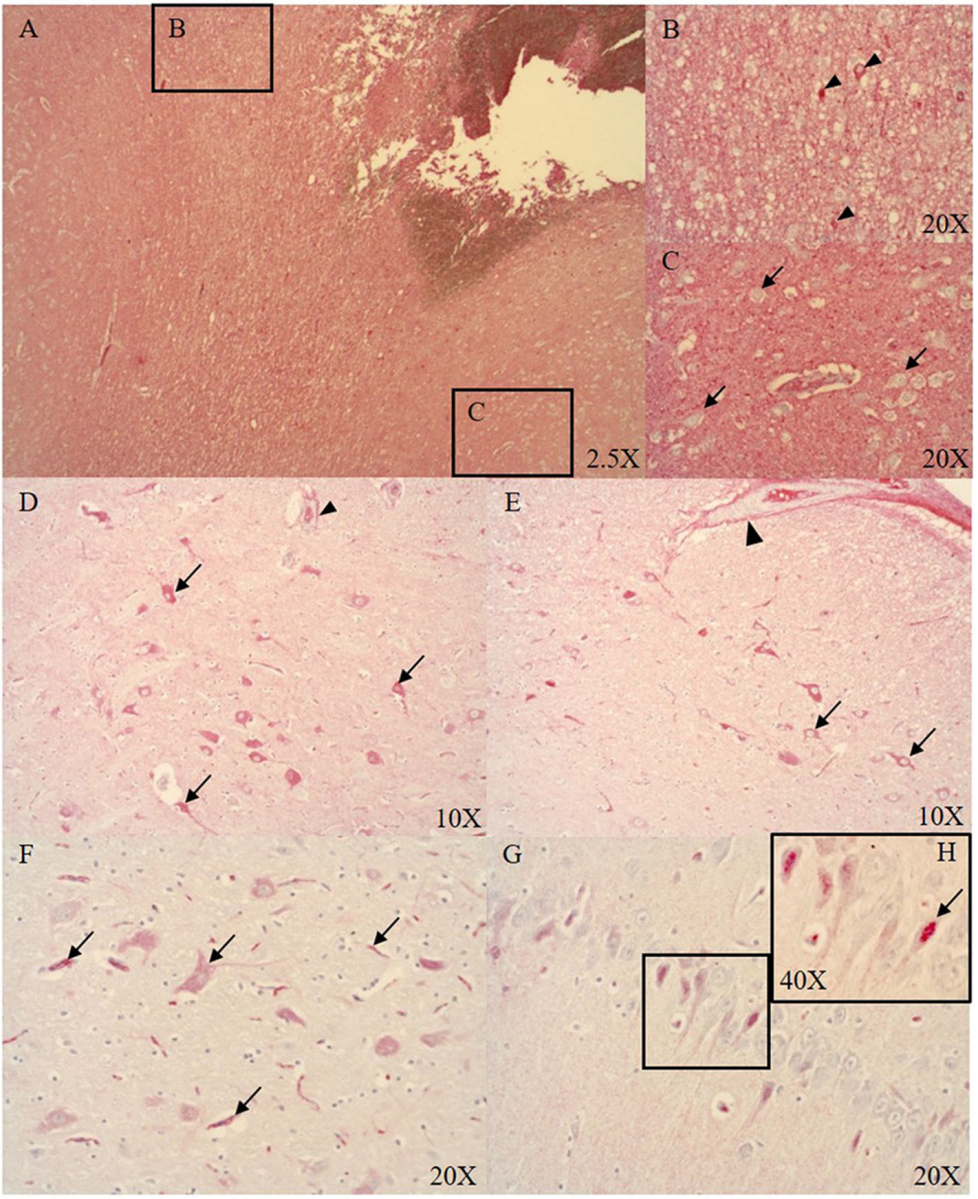
Alb expression in the ipsi- (**A–C**, midbrain cortical region) and contralateral (**D–G**, midbrain subcortical region) side (*n* = 9). Alb expression at the ASDH site (ipsilateral): extensive Alb extravasation **(A)**, singular neurons around the ASDH site are positive for Alb (black arrow heads **B**), most cortical neurons were negative for Alb (black arrows **C**). Alb expression in the contralateral hemisphere (distant to the ASDH site): minor Alb extravasation from microvasculature (indicated by positive adventitia of microvasculature (arrow head) and parenchyma), cytoplasmic positive neurons around vacuolization (black arrows **D**), pial artery SMCs negative, positive adventitia (arrow head), positive neurons associated with swelling (black arrows **E**), Alb contained in neurons and inside the microvasculature (black arrows **F**), hippocampal neurons show nuclear Alb expression **(G)** magnified in **(H)**, see black arrow. ASDH, acute subdural hematoma; Alb, albumin; SMCs, smooth muscle cells.

At the ASDH site, the parenchyma of necrotic areas with vacuolization (see Figure 2A), and singular neurons (see Figure 2B) were positive for Alb. However, the majority of the neurons were negative for Alb (see Figure 2C). Distant to the injury in midbrain subcortical regions, neurons were positive for Alb expression (see Figures 2D–F). Interestingly, in the hippocampus we found nuclear localization of Alb (see Figures 2G,H).

**Figure 3:**
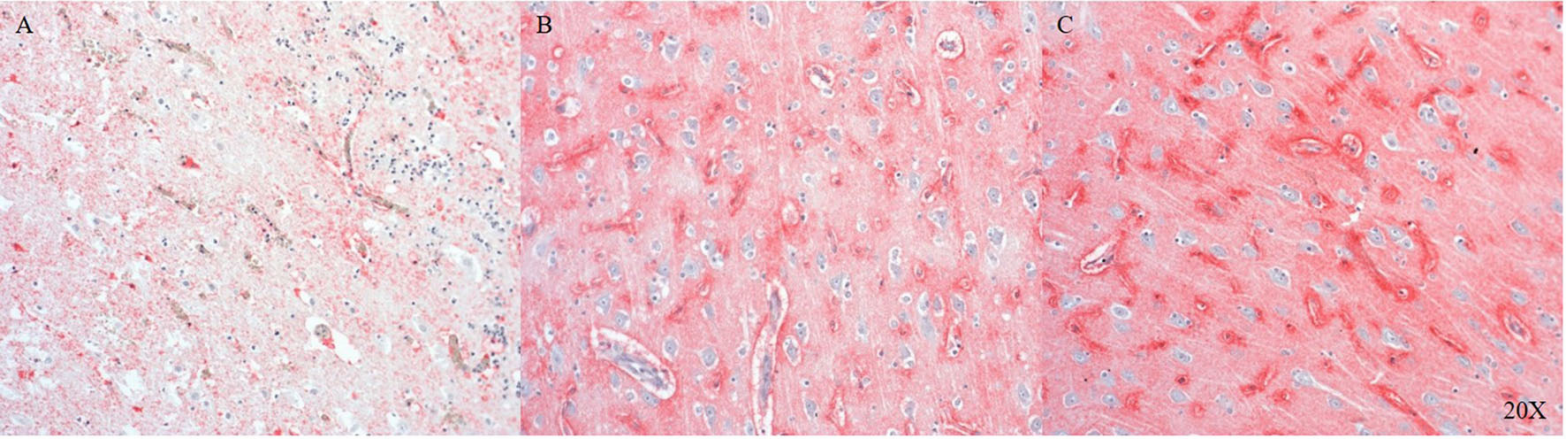
AQP4 expression in the parietal cortex at the injury site **(A)**, in neighboring areas **(B)** and distant to the injury site **(C)** (*n* = 6). At the injury site cellular architecture is impaired and AQP4 expression is weak **(A)**, moving away from the injury there is more AQP4 expression **(B)**, maximal AQP4 expression is present distant to the injury **(C)**. AQP4, aquaporin 4.

We established the staining of AQP4, a water channel protein, in the pig brain (parietal cortex). AQP4 shows variable expression as we move away from the injury site to the morphologically intact tissue (see Figures 3A–C).

**Figure 4:**
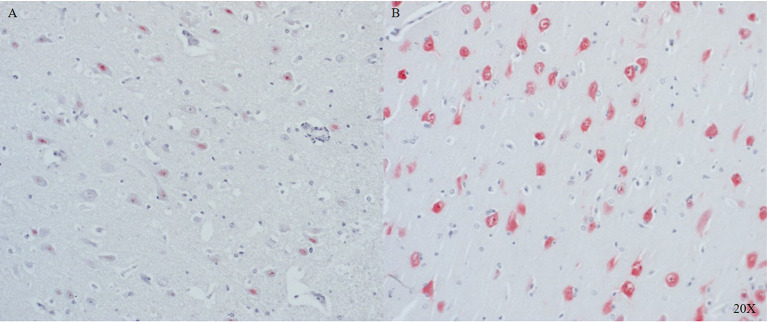
NeuN expression in cortical neurons of the frontal cortex close to **(A)** and further away **(B)** from the injury site (*n* = 6). Cortical neurons show little to no expression of NeuN in proximity to the injury **(A)**, in distance to the injury cortical neurons show NeuN expression **(B)**. NeuN, neuronal nuclei.

In the frontal cortex, NeuN was strongly expressed in the intact regions (see Figure 4B), and its expression was lower in neurons near the injury site (see Figure 4A).

### Vasculature

Pial arteries expressed OTR and OT in the smooth muscle cells (SMCs) and in the endothelium (see Figures 1A–D). Around pial and microvascular arteries there was a OTR and OT positive “blushing” expression pattern around sulci of the frontal cortex in the ipsilateral hemisphere (see Figures 1A,C). CSE was expressed in the SMCs of the pial arteries. In the microvasculature, CSE was expressed in vessel walls, mostly in the SMCs and the endothelium (see Figure 6B). In the microvasculature and pial arteries Alb was localized in the adventitia (see Figures 2D,E).

### Inflammation

**Figure 5:**
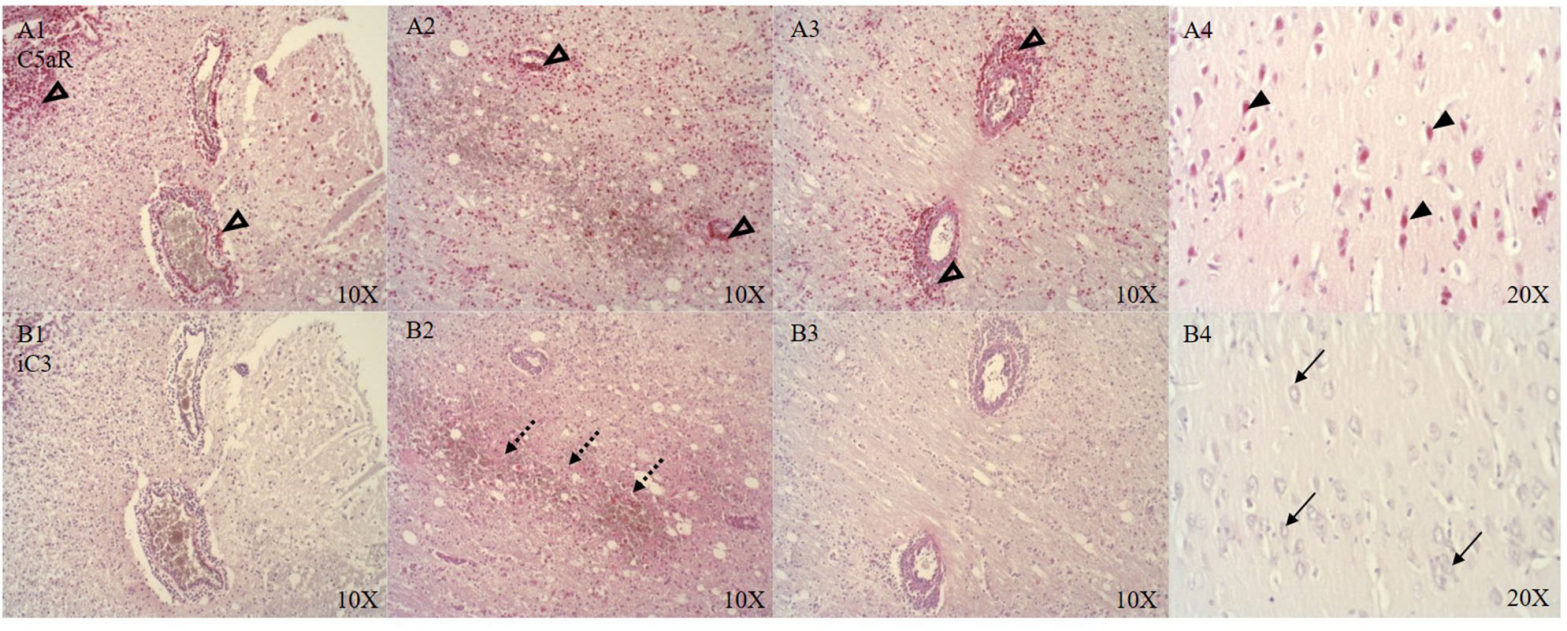
C5aR (**A**, *n* = 8) and iC3 (**B**, *n* = 7) expression around the ASDH site in four anatomical levels of the parietal cortex (A/B 1–3) and distant to the ASDH site (A/B 4). Around the ASDH site perivascular infiltration of C5aR positive immune cells (unfilled arrow heads **A1–3**), immune cell and parenchymal localization of iC3 (punctuated arrows **B1–3**), distant to the ASDH site nuclear expression of C5aR in cortical neurons (black arrow heads **A4**), no neuronal iC3 detection distant to the ASDH site (black arrows **B4**). C5aR, complement component 5a receptor; iC3, induced complement component 3; ASDH, acute subdural hematoma.

**Figure 6:**
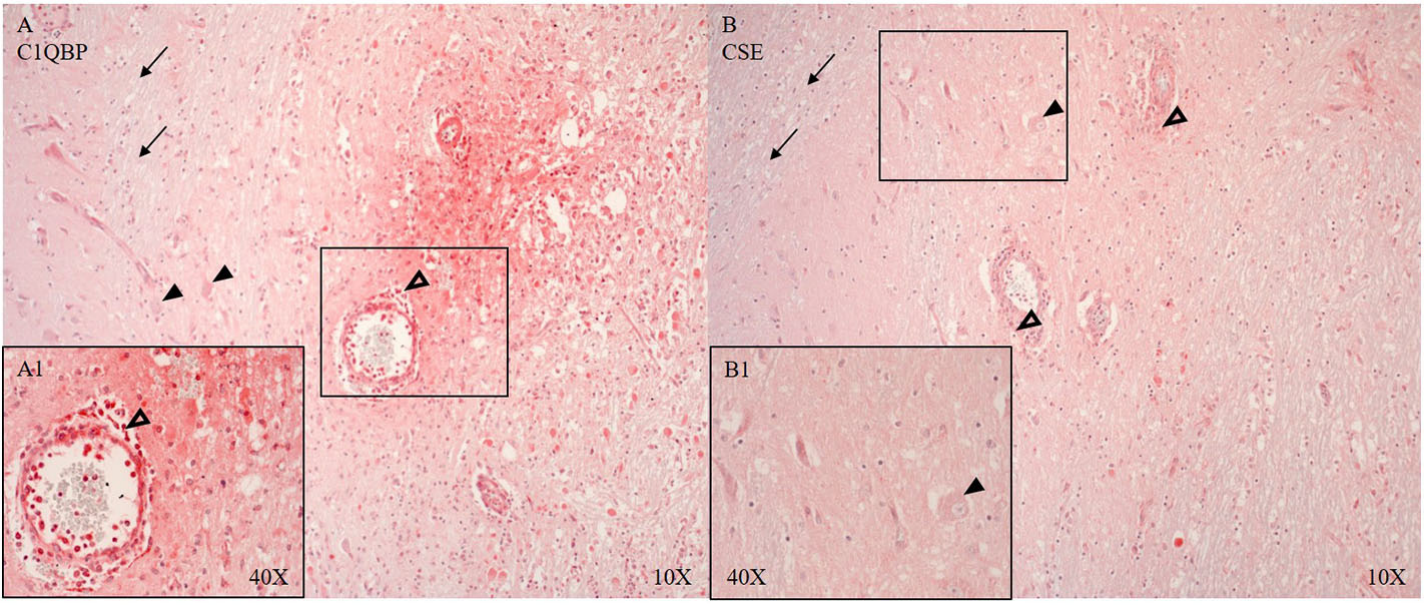
C1QBP (**A**, *n* = 5) and CSE (*B, n* = 7) expression in matching consecutive sections of the frontal cortex at the border zone of gray and white matter. Perivascular infiltration of C1QBP and CSE positive immune cells (unfilled arrow heads **A,A1,B**), C1QPB expression in the endothelium and SMCs of the microvascular artery **(A)** (vessel magnified in **A1**) and in the parenchyma with interstitial extravasated erythrocytes **(A,A1)**. C1QBP and CSE in neurons (black arrow heads) (**A,B** magnified in **B1**). CSE expression in the arterial SMCs. White matter is negative for both: C1QBP and CSE (black arrows) **(A,B)**. C1QBP, complement component 1 Q subcomponent-binding protein; CSE, cystathionine-ɤ-lyase; SMCs, smooth muscle cells.

CSE positive immune cells were present in and around the microvasculature and pial arteries (see Figure 6B). OTR positive immune cells were present near the ruptured microvasculature. Cortical neurons of the parietal cortex showed nuclear positivity for C5aR in proximity and distant to the ASDH site (see Figure 5A4). Close to the injury site there was an infiltration of C5aR-expressing immune cells in areas of intraparenchymal bleeding (see Figure 5A1–3). In the brainstem, C5aR was mostly present in the cytoplasm, in contrast to the cortical region. Expression of iC3 was more limited in the parietal cortex in comparison to C5aR but also found in infiltrating immune cells and parenchyma (see Figure 5B1–3). In the hippocampus, granular neurons also expressed C5aR. C1QBP was positive in the cytoplasm of neurons around the ASDH site in the frontal cortex (see Figure 6A). C1QBP was detected associated with the microvasculature, strongly expressed in immune cells within the vessel wall and in areas of discrete bleeding (see Figure 6A,A1).

**Figure 7:**
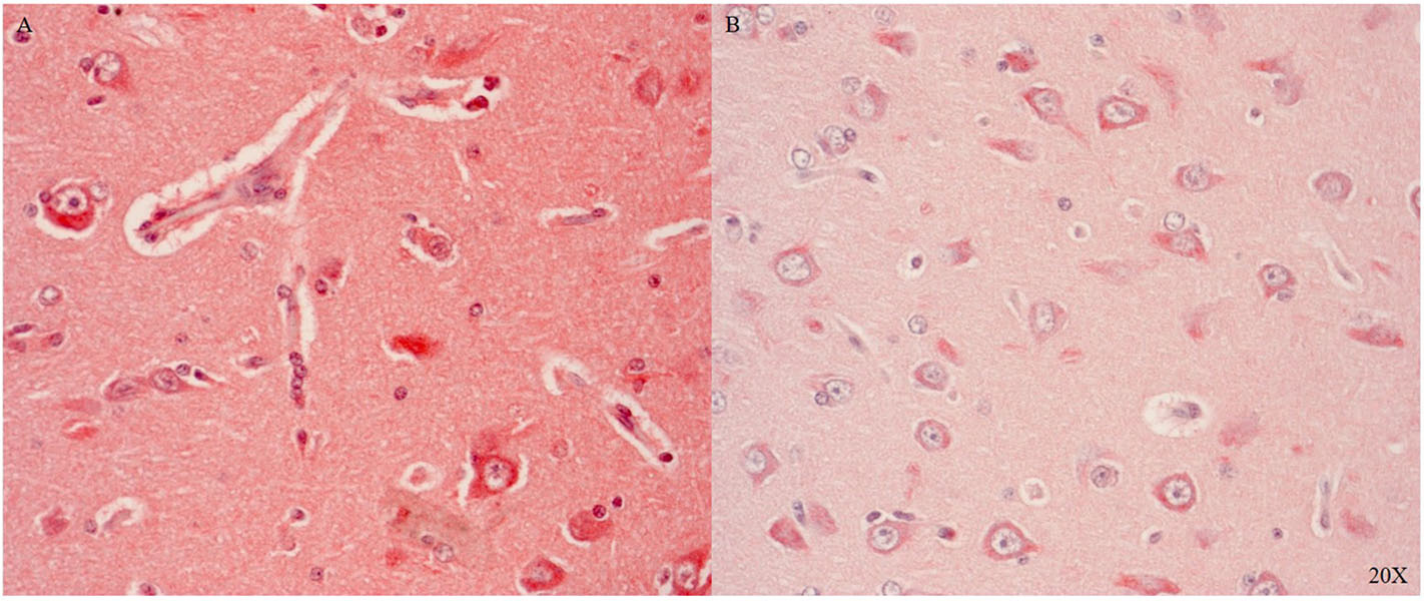
Il-6R expression in the parietal cortex in proximity **(A)** and distant to the injury **(B)** (*n* = 1). Il-6R expression in neurons, immune cells, parenchyma and the microvasculature close to the injury **(A)**, and limited neuronal cytoplasmic expression distant to the injury **(B)**. Il-6R, interleukin 6 receptor.

Il-6R is predominantly expressed in the parenchyma and in the cytoplasm of neurons in areas with perivascular swelling in the parietal cortex (see Figure 7A). Close to the injury, immune cells in the parenchyma and in the microvasculature were Il-6R positive (see Figure 7A). Further away from the injury Il-6R expression was limited to neuronal cytoplasmic expression (see Figure 7B).

**Figure 8:**
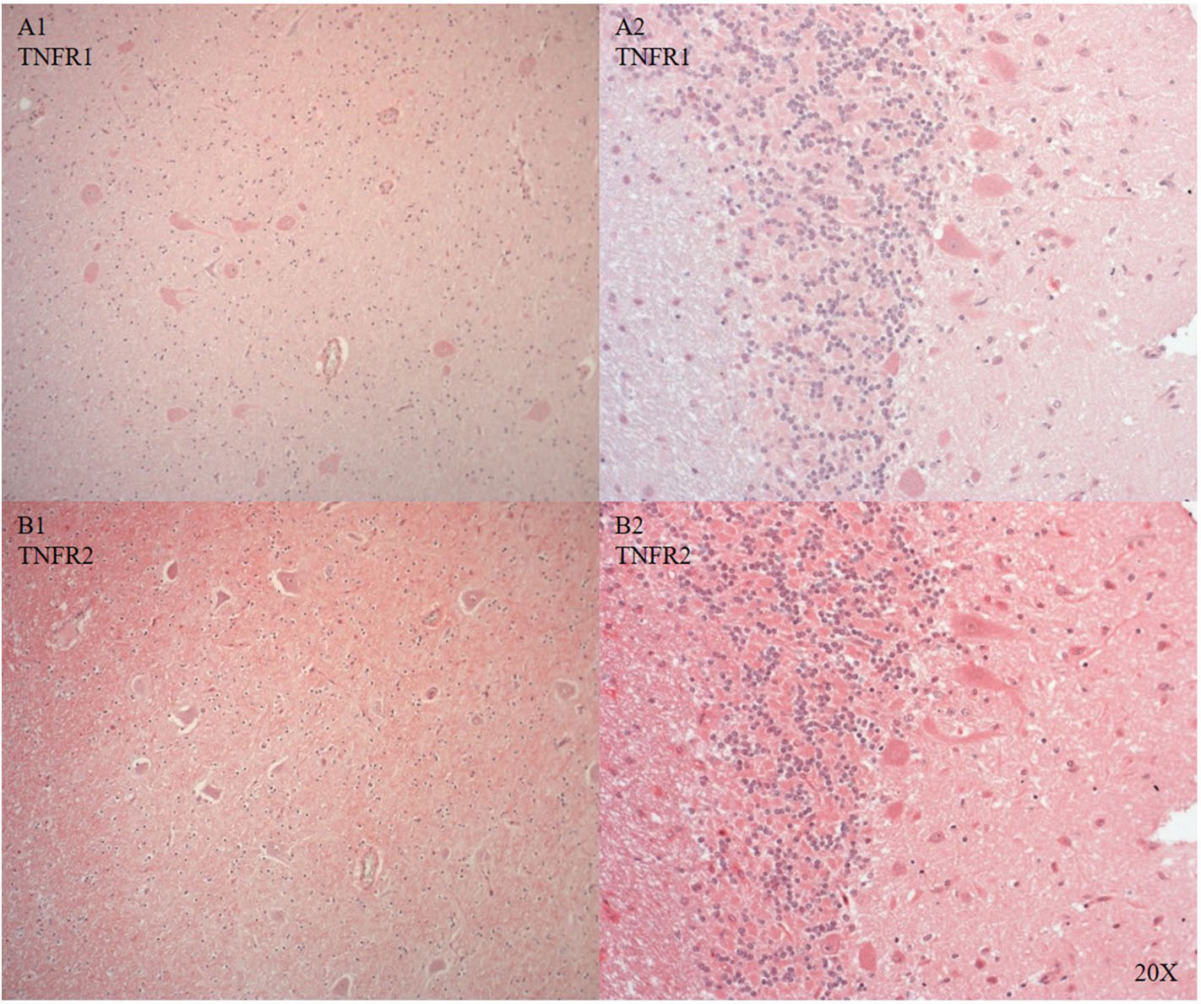
TNFR1 (**A**, *n* = 1) and TNFR2 (**B**, *n* = 1) in the brainstem (A/B 1) and in the cerebellum (A/B 2). In the brainstem TNFR1 **(A1)** and TNFR2 **(B1)** were expressed in neurons and microvasculature, and TNFR2 was also expressed in the parenchyma **(B)**. In the cerebellum purkinje cells expressed TNFR1 **(A2)** and TNFR2 was expressed in purkinje, granular cells and parenchyma **(B2)**. TNFR1, anti-tumor necrosis factor receptor 1; TNFR2, tumor necrosis factor receptor 2.

We only looked for TNFR1 and TNFR2 expression in the brainstem and the cerebellum, where the neurons showed cytoplasmic and nuclear TNFR1 and TNFR2 expression (see Figures 8A1,2,B1,2).

## Discussion

In available brain tissue from a previous study describing a porcine ASDH model, we localized H_**2**_S producing enzymes, as well as OTR and OT concurrent with markers of barrier dysfunction and local inflammation. The main findings were that CSE, CBS, OTR, and OT expression in the porcine brain were localized to: (i) cortical neurons in the gyri, (ii) in the parenchyma at the base of the sulci, (iii) microvasculature and pial arteries, and (iv) resident and infiltrating immune cells.

The cortical neurons in the gyri were positive for CSE (see Figure 1F1), but its expression was reduced in the ipsilateral side (see Figure 1E1), which may be related to the increased ICP. CSE was expressed and evenly distributed in the parenchyma of the contralateral side (see Figure 1F) and apparently lower in the ipsilateral side (see Figure 1E). CBS showed an opposite pattern and was not present in the contralateral hemisphere (see Figure 1H) but upregulated with injury immediately around the sulcus in the ipsilateral hemisphere (see Figure 1G), potentially due to the fact that the high ICP elicits the most stress at the base of the sulcus (2). This finding is particularly salient with regards to translational medicine, because in the gyrencephalic brain the pressure-induced injury is expected to be found distant to the surface at the depth of the sulci (2). This might also be related to a lack of tissue oxygenation, which commonly first occurs at the base of sulci, as observed in humans (25, 26). We see the same spatial reaction to injury found in humans in our pig model thus suggesting it may be an adequate model to study human brain injury (Figure 1). As shown in the inserts with high magnification of Figure 1, we observed perivascular swelling in both hemispheres, which agrees with TBI findings in humans showing “surrounding vasogenic edema within bilateral regions” (27).

Reports found in the literature on cerebral CSE expression are limited and ambiguous. Neuronal CSE mRNA and protein expression are reported in the cerebral cortex, in granular and purkinje cells in the cerebellum, and in pyramidal neurons in the hippocampus (28–30). In accordance with these findings, we detected CSE protein expression in hippocampal and cortical neurons in the contralateral hemisphere (see Figure 1F), in cerebellar punkinje cells and granular neurons. To our knowledge, this is the first report on cortical parenchymal CSE expression in gyri with constitutive expression in intact brain regions and reduced at the site of injury. CBS on the other hand, appears to be upregulated with injury (see Figure 1G). Its presence could not be detected in neurons (see Figures 1G1,H1), was variable in astrocytes and closely associated with the microvasculature, which is supported by the reports from the protein atlas (28, 31). In contrast to our findings, neuronal CBS expression has been reported in uninjured piglets, which may be more reflective of the developmental stage, whereas here, we analyzed adult pigs (32, 33). In fact, a study in humans revealed cerebral CBS expression pattern changes depending on age and neuronal damage (34).

We have reported the interaction of H_2_S and the OTR in trauma (8) and H_2_S can directly stimulate OT release on the hypothalamic level during fluid shifts and osmotic challenges (7, 35). OTR was abundantly expressed and OT less pronounced in cortical neurons and parenchyma around the base of the sulci, with increased expression in the ipsilateral side (see Figures 1A,C). The presence of OTR has been shown previously in human cortical neurons, in vascular profiles and around micro-infarcts in the gray and white matter, especially in activated astrocytes and vasculature (10, 36–38). This supports our findings of OTR and OT expression around the microvasculature in the ipsilateral hemisphere (see Figures 1A,C). These findings well agree with our previous results of the interaction between H_2_S and the OT/OTR systems (8, 39): OTR and CBS were both upregulated as a response to pressure induced injury, whereas CSE showed the inverse response. Noteworthy, CSE, CBS, OTR and OT, all showed expression at the base of the sulci in the ipsilateral side, where maximal pressure-induced stress occurs in the gyrencephalic brain (see Figure 1).

Administration of H_2_S has improved barrier integrity and reduced cerebral edema, and the loss of CSE expression has previously been associated with Alb extravasation and barrier dysfunction (11, 24, 40–42). Thus, the reduction of CSE on the ipsilateral side (see Figure 1E) might contribute to barrier dysfunction as evidenced by increased Alb extravasation (1).

Blood-borne protein and Alb uptake after BBB disruption has been shown to be accomplished by both astrocytes and neurons (43, 44). The presence of cerebral Alb leads to neuronal glutamate release into the extracellular matrix (43). Glucose and lactate levels are elevated as a consequence of injury and need to be cleared. Astrocytic glucose uptake and clearance is promoted by glutamate release from neurons which stimulates astrocytes to re-establish the extracellular milieu after trauma (45–47). We observed very few Alb positive neurons in the region of injury (see Figures 2A,B), and the majority of the cortical neurons in this area were negative for Alb (see Figures 2A,C). Interestingly, neuronal Alb was mostly present in the cytoplasm of morphologically normal cortical neurons distant to the ASDH (see Figures 2D–F). Intriguingly, in the hippocampus we found nuclear Alb staining in the neurons (see Figures 2G,H). Endogenous Alb production in the brain has been reported previously in response to injury, and is suggested to play a neuro-protective role (48, 49).

One of the limitations of the porcine model is the lack of established available biomarkers. The following are published key markers for the pig brain: 3-nitrotyrosine, NADPH oxidase subtype 2, glial fibrillary acidic protein (GFAP), ionized calcium-binding adapter molecule 1, microtubule-associated protein 2, β amyloid precursor protein, trefoil factor 3, brain-derived neurotrophic factor, Alb, CSE, CBS, OTR, and OT (1, 17–20, 32, 33, 37, 38, 50–52). Thus, we investigated the following additional markers to further characterize the ASDH model: AQP4, NeuN, C5aR, iC3 C1QPB, Il-6R, TNFR1, and TNFR2. The immunological markers were of particular interest because of the high homology of the innate immunity of the pig to the human: pigs are closer to the humans in 80% of the immune parameters investigated compared to rodents (53, 54).

AQP4 is the most abundant water channel protein in the human central nervous system (CNS) and is involved in edema and fluid homeostasis (55). We detected AQP4 in the pig brain and found that its expression was dependent on tissue integrity, declining with the severity of the injury and most abundant in intact tissue (see Figure 3).

NeuN was also found in the pig brain and serves as specific marker for post-mitotic neurons. The pattern of expression was similar to that described in humans after TBI wherein the strongest staining was found in regions with preserved cyto-architecture (see Figure 4) (56). To the best of our knowledge, this study is the first to identify porcine neurons with the neuronal marker NeuN.

Complement activation has been reported in CNS inflammation after acute brain injury, immune-mediated secondary neuropathology and BBB dysfunction (57–59). We detected C5aR (see Figure 5A1–3) positive immune cells and expression around the microvasculature which has been implicated in BBB dysfunction (60). We identified C5aR positive cortical neurons distant to the injury site (see Figure 5A4) which is in line with the literature on TBI (58), but there was no iC3 in neurons (see Figure 5B4). Presence of iC3 was detected in the parenchyma (see Figure 5B2) and around the immediate ASDH induction site, indicating complement activation.

C1QBP and CSE are shown around the ASDH induction site (see Figures 6A,B). The loss of CSE and the positive expression of C1QBP around the vessels (see Figure 6) may indicate a loss of the inhibitory function of H_2_S on C1QBP (61).

Il-6R, a pro-inflammatory marker, was expressed in immune cells in the cerebral vasculature and neurons after injury (see Figures 7A,B) confirming the literature (62–64). We investigated two additional inflammatory markers: TNFR1 and TNFR2. Both antibodies tested worked in the pig brain: brainstem and cerebellum showed differential cytoplasmic and nuclear neuronal expression (see Figures 8A1,2,B1,2).

The purpose of the current study was to describe protein expression patterns in the porcine brain in a clinically relevant resuscitated ASDH model. An analysis of the functional consequences of the ASDH in this model has been reported in a previous publication (1). Any causal link between H_2_S, OT/OTR and brain physiology, oxidative stress and inflammation was beyond the scope of the present study.

## Conclusion

The H_2_S, and the OT/OTR systems were shown to be expressed in the brain in a long-term, resuscitated porcine model of ASDH. In particular, they were identified in neurons, vasculature and parenchyma at the base of sulci, where pressure-induced injury leads to maximal stress in the gyrencephalic brain. We observed the same spatial relation to injury known for humans in our pig model suggesting it may be an appropriate model to study human brain injury. Cytoplasmic and nuclear presence of Alb was detected in morphological uninjured neurons distant from the ASDH induction site, which suggests an undescribed role for Alb after acute brain injury. We identified novel mediators of injury that can be used in the pig brain to further characterize the pathophysiology of ASDH and potential therapeutic approaches.

## Data Availability Statement

The raw data supporting the conclusions of this article will be made available by the authors, without undue reservation, to any qualified researcher.

## Ethics Statement

This study was approved by the Regierungspräsidium Tübingen (Germany) for Animal Research and the local Animal Care Committee (Reg.-Nr. 1316, date of approval June 20, 2016).

## Author Contributions

ND performed the immunohistochemistry, data analysis and interpretation, and drafting of the manuscript. SU performed the immunohistochemistry. AH performed animal experiments and removal of the brain during the necropsy. AS performed the neuropathological evaluation and together with TM, EN, and MH-L helped with input for analysis of markers, data interpretation, critical comments, and expert feedback on the manuscript. CW and HG contributed critical comments and expert feedback on the manuscript. OM contributed to the experimental design, supervising immunohistochemistry, data interpretation, and writing of the manuscript. PR contributed to the study design and edited and approved the final version of the manuscript. All authors read and approved the final version of the manuscript.

## Conflict of Interest

The authors declare that the research was conducted in the absence of any commercial or financial relationships that could be construed as a potential conflict of interest.
