# Supplementary material for: Cerebral Immunohistochemical Characterization of the H2S and the Oxytocin Systems in a Porcine Model of Acute Subdural Hematoma
Source: Front Neurol. 2020 Jul 7;11:649. doi: 10.3389/fneur.2020.00649 (PMC7358568; doi:10.3389/fneur.2020.00649)
Supplement: Supplementary file 1 [file Data_Sheet_1.pdf]

Supplemental Material

Material and Methods

Immunohistochemistry (IHC) control experiments

All primary antibodies were titrated to their optimal dilutions within the range recommended by the manufacturer. To check the specificity of IHC, we routinely perform control experiments. Whenever the immunogen peptide is available, we use primary antibody pre-incubated with the immunogen peptide 10X excess (3 h at room temperature or overnight at 4 °C) and incubate the tissue with this pre-absorbed antibody instead of primary antibody alone. A second negative control is performed via incubation with diluent instead of primary antibody to control for the specificity of the secondary system. Exemplary pictures of the performed negative controls for the CSE antibody are shown in Fig. S1.

Blocking of the slides with 10 % serum of the secondary antibody species before the primary antibody incubation prevents non-specific protein-protein interactions and cross-reactions with tissue Fc receptors. Non-specific activity of the alkaline phosphatase detection system is prevented by the addition of levamisole, which blocks all endogenous alkaline phosphatases except the intestinal isoform, which the detection is based on.

Oxytocin receptor immunoprecipitation

The specificity of the used oxytocin receptor (OTR) antibody from proteintech was confirmed in protein lysates from Caco2 cells, since this cell line was easily accessible to us and its expression and activity of OTR is reported in the literature (Klein et al. 2011, Journal of Cellular Biochemistry 112: 3216-3226). The cells were bred to 100 % confluency in T25 cell culture flasks, washed with PBS, and lysed with 100 µl Pierce RIPA buffer + protease inhibitor cocktail (Thermo Scientific) per flask. Cells were broken up with two freeze-thaw cycles in liquid nitrogen. After 90 min incubation on ice, the lysates were centrifuged for 20 min at 12000 rpm (4 °C) and the pellet was discarded. The samples for immunoprecipitation were prepared according to the manufacturer's instructions for the Pierce™ Protein-A/G-Agarose Kit (Thermo Scientific). Briefly, the lysate supernatant was diluted 1:1 in binding buffer, OTR antibody was added at a concentration of 5 µg/ml and incubated overnight (4°C). We used two different antibodies for the immunoprecipitation (IP), rabbit polyclonal (Proteintech) and mouse monoclonal (C-4, Santa Cruz). The IP samples were purified using the Pierce™ Protein-A/G-Agarose Kit (Thermo Scientific) and a buffer exchange to PBS was performed with Amicon Ultra 15 mL Centrifugal Filters (Merck Millipore) according to the manufacturer's instructions. The samples were analyzed via SDS-PAGE and Western blot detection was performed with the rabbit polyclonal OTR antibody (1:1000, Proteintech).

Results

IHC control experiments

The pictures in Fig. S1 B/B1 and C/C1 show exemplary IHC negative control experiments for CSE. A positive signal could be prevented by either pre-incubation of the primary antibody with the immunogen peptide (B/B1) or performing the IHC with diluent without the addition of primary antibody, suggesting that the positive red signal in Fig. S1 A/A1 (replicated from Fig. 1 E/E1) is specific for CSE.

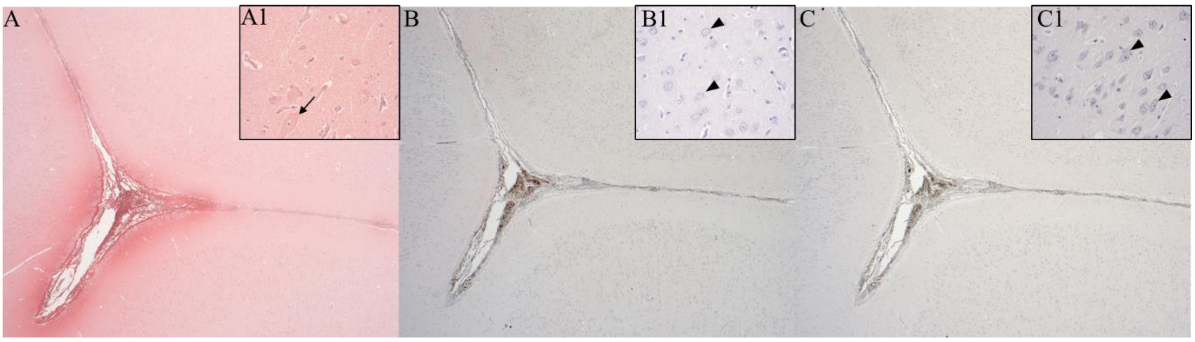

Figure S1: Consecutive tissue sections of CSE immunohistochemistry and negative controls, (A, B, C) 2.5X overview pictures, inserts (A1. B1, C1) 40X magnification. (A/A1) are a copy of Fig. 1E/E1 as an example of positive CSE (CSE ab: 12217-1-AP Proteintech) IHC, with the arrow in A1 indicating a neuron with little CSE expression (pink) and nuclear hematoxylin counter-staining. (B/B1) are examples of IHC with pre-absorbed (CSE fusion protein: Ag2872 Proteintech) primary antibody, arrows in B1 pointing to negative neurons with nuclear hematoxylin counter-staining. (C/C1) are examples of IHC with diluent without primary antibody, arrows in C1 pointing to negative neurons with nuclear hematoxylin counter-staining.

Oxytocin receptor immunoprecipitation

As can be seen in Fig. S2, the IP of OTR from Caco2 cells with either the monoclonal or polyclonal OTR antibody detected the polyclonal OTR antibody revealed a single band for each set of samples. Thus, we concluded that both antibodies specifically detect OTR. We used the polyclonal antibody for our experiments, since the monoclonal antibody neither was validated nor did it work for IHC on paraffin embedded tissue.

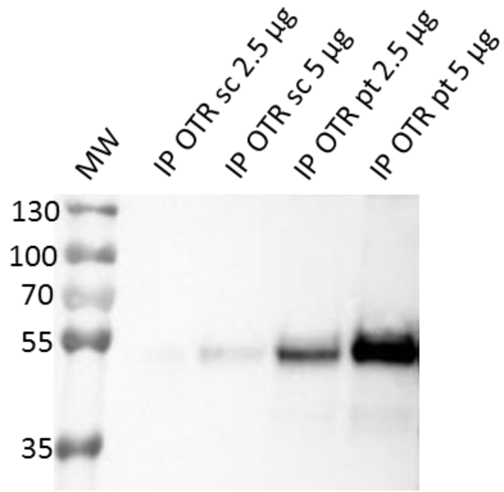

Figure S2: Western blot of oxytocin receptor (OTR) immunoprecipitation (IP) of Caco2 cells. Sample loading is indicated above the blots: antibody used for IP and amount of protein loaded. MW = molecular weight marker, sc = Santa Cruz, pt = Proteintech. The blot was detected with polyclonal OTR antibody (1:1000, Proteintech).
